# Performance of seven commercial automated assays for the detection of low levels of anti-*Toxoplasma* IgG in French immunocompromised patients

**DOI:** 10.1051/parasite/2019052

**Published:** 2019-08-23

**Authors:** Tiphaine Douet, Catherine Armengol, Elena Charpentier, Pamela Chauvin, Sophie Cassaing, Xavier Iriart, Antoine Berry, Judith Fillaux

**Affiliations:** 1 Service de Parasitologie – Mycologie, Centre Hospitalier Universitaire de Toulouse-Purpan 330 avenue de Grande Bretagne 31059 Toulouse France; 2 Laboratoire d’analyse biomédicale, Centre Hospitalier Comminges Pyrénées Avenue de Saint Plancard 31806 Saint Gaudens France; 3 Pharmacochimie et Biologie Pour le Développement (PHARMA-DEV), IRD UMR 152 Université Paul Sabatier 35 Chemin des Maraîchers 31400 Toulouse France

**Keywords:** *Toxoplasma gondii*, IgG low level, Immunodiagnosis, Automated tests

## Abstract

*Background*: Immunocompromised patients are at high risk for the development of severe toxoplasmosis from tissue cyst reactivation, the most frequently, or from recently acquired acute infections. Knowledge of serologic status is therefore crucial. Screening for toxoplasmosis is sometimes performed while patients are already immunocompromised and have a low or even undetectable IgG titer by routine automated enzyme immunoassays. The aim of this study was to assess the sensitivity and specificity of seven reagents for the detection of low levels of IgG. Sera from 354 patients were collected and analysed. *Results*: Elecsys^®^ offered the best analytic performances, superior to those of Architect^®^ and Platelia^®^, which were superior to those of Access II^®^ and TGS TA^®^. Vidas II^®^ and Liaison II^®^ reagents exhibited poor analytical performances in this cohort. For Elecsys^®^, Platelia^®^ and Architect^®^, new thresholds for the grey zone and positive zone have been defined to improve the sensitivity of these reagents while maintaining excellent specificity. *Conclusions*: Commercialized assays for toxoplasmosis screening are not suitable for IgG low-level detection in patients without adapting the supplier thresholds to avoid false negative results and risk generalized toxoplasmosis.

## Abstract (fr)

*Contexte* : Les patients immunodéprimés courent un risque élevé de développer une toxoplasmose grave résultant de la réactivation de kystes tissulaires, le plus souvent, ou d’infections aiguës récemment contractées. La connaissance du statut sérologique est donc cruciale. Le dépistage de la toxoplasmose est parfois effectué chez des patients déjà immunodéprimés et dont le titre en IgG est faible, voire indétectable, par les immunoessais enzymatiques automatisés de routine. Le but de cette étude était d’évaluer la sensibilité et la spécificité de sept réactifs pour la détection de faibles taux d’IgG. Les sérums de 354 patients ont été recueillis et analysés. *Résultats* : Elecsys^®^ offre les meilleures performances analytiques, supérieures à celles d’Architect^®^ et de Platelia^®^, supérieures à celles d’Access II^®^ et de TGS TA^®^. Les réactifs Vidas II^®^ et Liaison II^®^ ont présenté des performances analytiques médiocres dans cette cohorte. Pour Elecsys^®^, Platelia® et Architect^®^, de nouveaux seuils pour la zone grise et la zone positive ont été définis pour améliorer la sensibilité de ces réactifs tout en maintenant une excellente spécificité. *Conclusions* : Les tests commercialisés pour le dépistage de la toxoplasmose ne conviennent pas à la détection de faibles taux d’IgG chez les patients sans adaptation des seuils des fournisseurs pour éviter les résultats faux négatifs et le risque de toxoplasmose généralisée.

## Introduction

Toxoplasmosis is a zoonotic infection that may cause a large spectrum of clinical diseases. Generally asymptomatic, infection with *Toxoplasma gondii*, a cosmopolite protozoan parasite, leads to differentiation into latent bradyzoite forms within tissue cysts that persist indefinitely throughout the life of the host. In immunocompromised patients, recently acquired acute infection or tissue cyst reactivation, the most frequently, can cause severe toxoplasmosis and may lead to fatal outcomes if not correctly treated [18]. These patients are usually tested for the presence of IgG antibody against *T. gondii*. In the case of a positive result or an association of a negative result and a positive donor, antitoxoplasmosis prophylactic therapy is prescribed to avoid severe infection or reactivation [10, 14]. Screening for toxoplasmosis is sometimes performed when patients are already immunocompromised and have a low or even undetectable IgG titer by routine automated enzyme immunoassays. These tests are used for the diagnosis of toxoplasmosis in the general population but especially in at-risk populations such as pregnant women and immunodepressed patients. Most manufacturers promote the specificity of their tests as having a very low false-positive number and avoid considering a pregnant woman as positive and subsequently not receiving monthly monitoring during pregnancy. For immunocompromised patients, it is important to have a very sensitive serological test for IgG to avoid false negative serology while the patient presents a very low IgG level. The results are often equivocal, regardless of the technique used, when the IgG concentrations are close to the threshold value of the assay. In these situations, performing a second technique is recommended even though, in most cases, the immune status of the patient remains doubtful. A confirmatory test is then necessary to unambiguously determine the patient’s serological status [6]. The objective of our study was to evaluate the sensitivity and specificity of seven automated assays for the detection of anti-*T. gondii* IgG in patients with low antibody levels and propose a new screening threshold depending on the technique used.

## Materials and methods

### Ethical considerations

The routine diagnostic methods were used during routine laboratory work-up for the patients who received written laboratory reports. The evaluated diagnostic methods made use of excess serum. Patient characteristics were obtained from a non-interventional review of medical charts and laboratory results. According to French law, the patients were informed and retained the right to oppose the use of their anonymised medical data for research purposes. Dedicated ethical approval and individual patient consent were not necessary for this type of study [1, 2].

### Sample collection

Patients from transplantation or haematology units were included if they presented low or negative titres of IgG, without IgM, when screened for toxoplasmosis in the Parasitology and Mycology Unit of the Toulouse University Hospital, from 1st January to 31st December 2016. If duplicate, only one serum per patient was included in the study.

### Laboratory investigation

#### Routine diagnostic methods

Sera were prospectively assessed with the Architect Toxo IgG^®^ and Architect Toxo IgM^®^ assays on an automated analyser Architect i2000 (Abbott Laboratories, Wiesbaden, Germany). In the case of a non-positive titre of IgG, i.e., less than 3 IU/mL, a Platelia Toxo IgG^®^ test on an automated Evolis analyser (BioRad, Marnes-La-Coquette, France) was performed. If the results were discrepant between the screening assays, an LDBio-Toxo II IgG^®^ Western blot (WB) assay (LDBio, Lyon, France) was performed to confirm the presence or absence of specific IgG.

Samples were frozen at −20 °C until further analyses. For Platelia^®^ and LDBIO II^®^, missing data were completed retrospectively.

#### Evaluated diagnostic methods

The analyses with the Vidas Toxo IgG II^®^ assay on an automated Mini-Vidas analyser (BioMérieux, Marcy l’Étoile, France), Liaison Toxo IgG II^®^ assay on an automated Liaison XL analyser (DiaSorin, Saluggia, Italy), Elecsys Toxo IgG^®^ assays on an automated Cobas 8000 analyser (Roche Diagnostics, Mannheim, Germany), Access Toxo IgG II^®^ on an automated Access analyser (Beckman Coulter Inc), and the TGS TA Toxo IgG^®^ assays (TGS Technogenetics, Milan, Italy) on an automated IDS-iSYS system (Immunodiagnostic Systems, Boldon, UK) were performed retrospectively, from January 2017 to December 2017. Except for the Access, which was located in the Saint Gaudens Regional Hospital Centre, all the sera were analysed in the medical analysis laboratory of the Toulouse University Hospital.

All tests were performed as instructed by the manufacturers, with an identical independent control protocol, under the supervision of two certified biologist in Parasitology. The cut-off values for IgG detection used to interpret the results were those recommended by the manufacturers (Table 1). All immunoassays reported the test results in IU/mL, except for LDBIO II^®^.

**Table 1:** IgG cut-off values recommended by the manufacturers.

Assays/system	Technique	WHO IS	Negative	Gray zone	Positive
Elecsys/Cobas 8000	ECLIA recombinants SAG1 (P30)	3rd sera (TOXM)	<1	1 ≤ *x* < 30	≥30
Architect/i2000	CMIA recombinants SAG1 (P30) GR8	1st IgG (01/600)	<1.6	1.6 ≤ *x* < 3	≥3
Platelia/Evolis	ELISA inactivated *T. gondii*	3rd sera (TOXM)	6	6 ≤ *x* < 9	≥9
Access II/Access	CLIA inactivated *T. gondii*	3rd sera (TOXM)	<7.5	7.5 ≤ *x* < 10.5	≥10.5
TGS TA/IDS-iSYS	CLIA purified *T. gondii*	Unknown	<1.5		≥1.5
Vidas II/Mini Vidas	ELFA inactivated *T. gondii*	2nd sera (TOXS)	<4	4 ≤ *x* < 8	≥8
Liaison II/Liaison XL	CLIA inactivated *T. gondii*	2nd sera (TOXS)	<7.2	7.2 ≤ *x* < 8.8	≥8.8
LDBio II	WB P30 P31 P33 P40 P45	NA	<3 bands or no 30 kDa band		≥3 bands including the 30 kDa band

WHO IS, World Health Organization International Standard; NA, not available.

### Statistical analysis

The characteristics of the studied population were described using percentages and medians along with interquartile ranges instead of means and standard deviations when distributions were found to be non-Gaussian. Screening tests were evaluated against the confirmatory test (Western blot LDBio II). A sensitivity and specificity calculation was performed on all the sera included in the study. The analysis of the results was performed using Receiving Operating Characteristic (ROC) curves to determine which threshold(s) would be most suitable for the screening of patients with low levels of IgG for each test. The sensitivity and specificity results were compared using a test of equality of proportions. The areas under the curve were compared by a *χ* ^2^ test. The threshold of significance was set at 5%.

All statistical tests and procedures were performed using the Intercooled Stata 9.2 statistical package (StataCorp, College Station, TX, USA).

## Results

### Description of population, samples and assays

**Figure 1:**
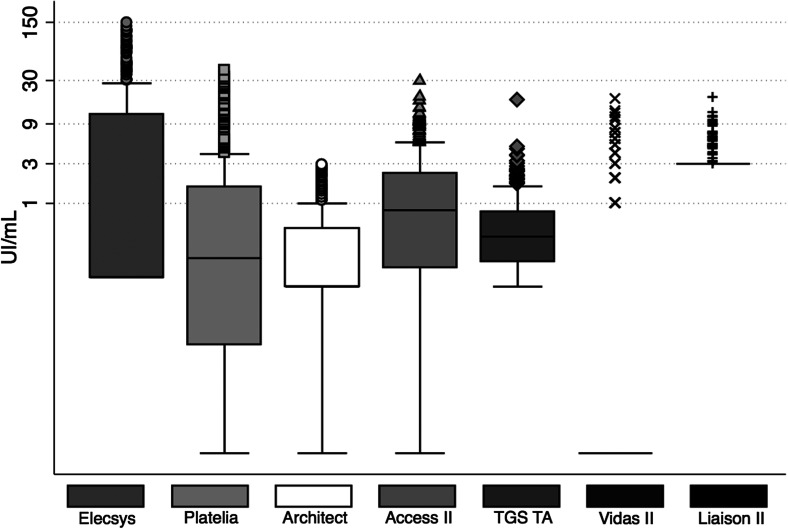
Distribution of the low IgG level sera according to the automated assays.

From 1st January to 31st December 2016, a total of 16,250 sera from 10,104 patients were tested for toxoplasmosis serology in the Toulouse University Hospital. Among these patients, 367 matched the inclusion criteria and were included in the study. The median age was 39 years (IQR, 23-56). The sex ratio was 1.2. All sera were assessed with Architect^®^, Liaison II^®^ and TGS TA^®^, 366 with Platelia^®^ and Vidas II^®^, 365 with Access II^®^, 356 with Elecsys^®^ and 360 with LDBio II^®^. Figure 1 shows the distribution of the range of IU/mL values according to the different methods for low level IgG.

Among these sera, a complete dataset was available for 354 samples. According to the Western blot results (study gold standard), 23.2% (82/354) of the patients presented serologies consistent with chronic toxoplasmosis infection. Table 2 shows the immune status of the population according to the studied assays.

**Table 2:** Number of patients not immunised, undefined and immunised according to manufacturers’ thresholds (number of LDBio II positive for each case, *N* = 82).

Assay/system	Non immunised (LDBio II+)	Undefined (LDBio II+)	Immunised (LDBio II+)
Elecsys/Cobas 8000	233 (0)	59 (22)	62 (60)
Architect/i2000	318 (46)	36 (36)	NA
Platelia/Evolis	324 (52)	3 (3)	27 (27)
Access II/Access	325 (55)	17 (16)	12 (11)
TGS TA/IDS-iSYS	307 (44)	NA	47 (38)
Vidas II/Mini Vidas	323 (51)	21 (21)	10 (10)
Liaison II/Liaison XL	343 (71)	2 (2)	9 (9)

NA, not available.

Of the 82 positive WB LDBio II, 91% (*n* = 75) of the sera had at least bands P30, P31 and P33. Among them, the profile with bands P30, P31, P33 and P40 was most often found (47%, *n* = 35). Of the 272 negative Western blots, 221 had no band, 33 had band P30 with less than two other bands, of which 18 had band P30 only.

### Sensitivity and specificity at the supplier thresholds

In Table 3, the specificity, sensitivity, positive predictive value (PPV) and negative predictive value (NPV) are presented for each reagent, with an estimated population seroprevalence of toxoplasmosis of 31.3% [13, 15, 17]. At first, the values in the grey zone were considered positive values, and these values were then included in the negative values. For TGS TA, which does not have a grey zone, there were no changes to the results. For Architect, since the samples were selected to be negative or in the grey zone, the calculation of the different values considering the sera in the grey zone as negative sera could not be performed. The specificity of Elecsys was significantly increased (*p* < 0.001) when the grey zone values were included in the negative values, and its sensitivity significantly increased when the grey zone results were considered positive (*p* < 0.001). Platelia, Architect, Vidas II, Liaison II and Access II had excellent 100% specificity regardless of the choice made for doubtful results. For Access II and Vidas II, the sensitivity of the test was significantly increased when the grey zone results were considered positive (*p* < 0.001). For Platelia and Liaison II, the sensitivity of the test was not significantly different according to the choice made for the grey zone results (*p* > 0.8).

**Table 3:** Relative sensitivity, specificity, PPV, and NPV by testing 82 positive and 272 negative samples for anti-toxoplasma IgG.^a^

Immunoassay and classification of equivocal result	Sensitivity^b^ (%) [95% CI]	Specificity^b^ (%) [95% CI]	PPV^c^ (%)	NPV^c^ (%)
Elecsys/Cobas 8000				
Positive	100 [100–100]	85.7 [82.0–89.3]	76.1	100
Negative	73.2 [68.7–77.8]	99.3 [98.4–100]	97.8	89.0
Architect/i2000				
Positive	43.9 [38.7–49.1]	100 [100–100]	100	79.6
Platelia/Evolis				
Positive	36.6 [31.6–41.6]	100 [100–100]	100	77.6
Negative	32.9 [28.0–37.8]	100 [100–100]	100	76.6
Access II/Access				
Positive	32.9 [28.0–37.8]	99.3 [28.4–100]	95.3	76.5
Negative	13.4 [9.7–17.0]	99.6 [99–100]	94.3	71.6
TGS TA/IDS-iSYS				
Positive/Negative	46.3 [41.1–51.5]	96.7 [94.8–98.6]	86.4	79.8
Vidas II/Mini Vidas				
Positive	37.8 [32.7–42.9]	100 [100–100]	100	77.9
Negative	12.2 [8.8–15.6]	100 [100–100]	100	71.4
Liaison II/Liaison XL				
Positive	13.4 [9.7–17.0]	100 [100–100]	100	71.7
Negative	11.0 [7.7–14.2]	100 [100–100]	100	71.1

PPV, positive predictive value; NPV, negative predictive value.

a The criteria were determined with the high and low cut-off values specified by the manufacturers. The confirmatory test was the Toxo II IgG Western blot (LDBio).

b Performance values were calculated using the Toxo II IgG test Western blot as the reference test.

c PPVs and NPVs were calculated using an estimated seroprevalence in France (31.3%).

### Analytical performance and adjustment of thresholds

**Figure 2:**
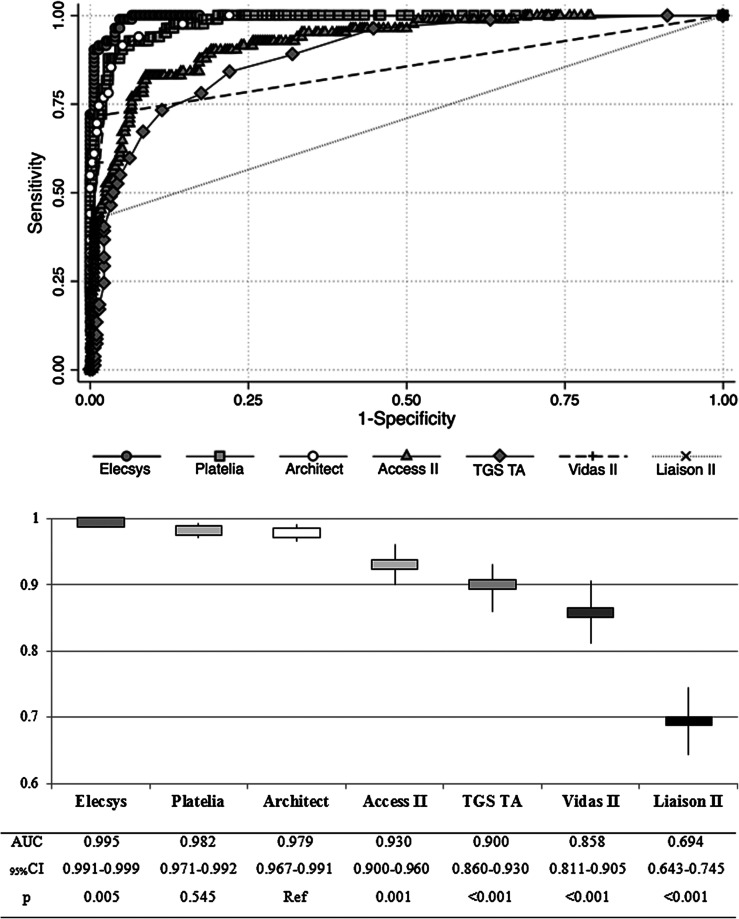
Areas under Receiving Operator Curves for each tested assay and comparison (*χ* ^2^ test) of analytic performances.

Five of the seven reagents tested were very informative. As shown in Figure 2, Elecsys had the largest AUC, which was significantly higher than that of Architect, our reference assay. Platelia’s performance was no different from that of Architect. Access II and TGS TA reagents, while acceptable, showed significantly lower performance than Architect. Vidas II and Liaison II were moderately informative and uninformative in the study population.

For Architect and Platelia, Table 4 presents the different thresholds that could be applied to the study population to improve diagnostic sensitivities and specificities, and thus to increase the number of correctly classified patients. For Elecsys (Table 4), we proposed to “tighten” the grey zone to maintain the same sensitivity but to improve the specificity of the test. Values close to the supplier thresholds are shown in bold and the proposed thresholds are highlighted. No threshold values could be proposed for the other reagents, to be both sensitive and specific enough not to need to carry out too many confirmatory tests.

**Table 4:** Sensitivities and specificities of Architect, Platelia and Roche according to the threshold.

Architect (threshold)	Sensitivity (%)	Specificity (%)	Platelia (threshold)	Sensitivity (%)	Specificity (%)	Elecsys (threshold)	Sensitivity (%)	Specificity (%)
0.00	100.00	0.00	0.00	100.00	0.00	0.13	100.00	0.00
0.30^a^	97.56	85.29	0.53	98.78	79.78	0.47	100.00	85.06
0.40	93.90	92.28	1.02^a^	95.12	88.24	1.12^c^	100.00	85.66
0.50	91.46	94.85	1.58	91.46	94.85	6.70^a^	100.00	93.38
0.90	74.39	98.53	2.43	78.05	98.16	9.43	98.78	95.22
1.00	69.51	98.90	4.76^b^	53.66	100.00	20.51^b^	90.24	99.26
1.40^b^	54.88	100.00	5.90^c^	37.80	100.00	30.13^c^	73.17	99.26
1.60^c^	43.90	100.00	9.09^c^	32.93	100.00	30.57	71.95	100.00

a New threshold for grey zone.

b New threshold for positive zone.

c Values close to the supplier thresholds.

### Sensitivities and specificities at the new thresholds

The concordance between the three reagents for which the thresholds were modified and the WB was 71.8% (254/354), significantly improved compared to the former thresholds (64.1%, *p* = 0.014). For Architect and Platelia (Table 5), the sensitivity was significantly improved when the threshold was changed (*p* < 0.001) but the specificity was significantly decreased (*p* < 0.001). For Architect, only two sera would have been falsely negative. A WB would be performed on 75 questionable results, of which 35 would be positive. The findings were similar for Platelia; four sera would have been falsely negative, and 66 WB will have to be made, of which 34 will be positive. These threshold changes made it possible to have very good sensitivity and to increase the VPN, but they led to an increase in the number of WB to be carried out. However, the grey zone was better defined, and these WBs were made more optimally. The new thresholds, therefore, allow Architect and Platelia to detect more positive sera. For Elecsys (Table 5), the contribution was quite different. The new thresholds allowed for better specificity while achieving fewer WBs. In fact, the sensitivity was 100% with the old thresholds, but by “tightening” its thresholds, this sensitivity can be preserved while decreasing the number of WBs to perform. By adjusting the entry threshold in the grey zone to 6.7 IU/mL, 254 sera were negative and confirmed, “saving” 21 WBs. The specificity of Elecsys was significantly increased (*p* < 0.001) when the grey zone entry threshold was increased from 1.00 to 6.70.

**Table 5:** Sensitivity, specificity, PPV, NPV of each reagent (former and new thresholds), 31.3% prevalence.

Assay/system	Sensitivity (%) [95% CI]	Specificity (%) [95% CI]	PPV (%)	NPV (%)
Elecsys/Cobas 8000				
Positive ≥ 1.00	100 [100–100]	85.7 [82.0–89.3]	76.1	100
Positive ≥ 6.70	100 [100–100]	93.4 [90.8–96.0]	87.3	100
Architect/i2000				
Positive ≥ 1.60	43.9 [38.7–49.1]	100 [100–100]	100	79.6
Positive ≥ 0.30	97.6 [95.9–99.2]	85.3 [81.6–89.0]	75.1	98.7
Platelia/Evolis				
Positive ≥ 6.00	36.6 [31.6–41.6]	100 [100–100]	100	77.6
Positive ≥ 1.02	93.9 [91.4–96.4]	89.0 [85.7–92.2]	79.5	97.0

PPV, positive predictive value; NPV, negative predictive value.

## Discussion

This study presents a comparison of the analytical performances of seven reagents for the detection of anti-*Toxoplasma gondii* IgG in a large cohort of low IgG level patients relative to a reference WB.

For diagnostic centres associated with clinical units that support immunosuppressed patients, Elecsys Toxo IgG^®^, Architect Toxo IgG^®^, Platelia Toxo IgG^®^, Access Toxo IgG II^®^ and TGS TA Toxo IgG^®^ appeared to be sufficiently informative to be routinely used for toxoplasmosis screening in patients with low IgG levels. However, Elecsys showed an analytic quality that was statistically superior to that of Architect or Platelia, which were superior to Access II and TGS TA. Vidas Toxo IgG II^®^ and Liaison Toxo IgG II^®^ showed poor analytical performance in this cohort. In all cases, the supplier thresholds did not seem optimal for this population and needed to be adapted by the user.

The limits of our study were the monocentric selection of sera on Architect to have only negative or doubtful sera. The exclusion of positive sera from our group of immunocompromised patients may artificially decrease the overall sensitivity of the different reagents.

In our study, a significant variation between the maximum levels of IgG was observed. This variation has been described in two other studies [9, 16]. The highest rates were always found with Elecsys. These differences in levels could be due to the composition of the antigenic solutions [19] or to the international standards chosen for calibration, although in the Maudry et al*.* study [9], no significant differences were shown. For all these reasons, patients are advised to be followed up in the same laboratory or, failing this, by the same screening technique.

The sensitivities of the tested assays were low, even when the doubtful values were included in the positive values, except for Elecsys. Patients with low IgG levels are often poorly represented in the studies that analyse and compare the different commercial assays. Leslé et al*.* [7] compared 231 doubtful or negative sera on Elecsys with Platelia results. For discordant results between the two reagents, a WB LDBio II was performed. In most cases, both techniques had discordant results (92.2%), indicating the need for a confirmatory test. Levigne et al*.* [8] compared TGS TA to Architect. Among the 21 discordant sera, 16 were made either negative or doubtful with Architect but were made positive with TGS TA, eight of which were confirmed positive with a second technique (Vidas, Axsym or Enzygnost). Data from these eight patients showed that this involved patients with transient infection and a low IgG level. TGS TA appeared to be more sensitive for the detection of low IgG levels at the supplier threshold. The study by Villard et al. [16] evaluated nine immunoenzymatic technologies: Advia, Architect, Axsym, Elecsys, Enzygnost, Liaison, Platelia, Vidas and Vidia. As in our study, Elecsys had excellent sensitivity, while that of Liaison was weak. However, we found Vidas and Platelia to have lower sensitivities. This may be due to the low number of sera examined in this study. The work of Franck et al. [4] evaluated the specificity and sensitivity of WB LDBio II as a confirmatory test for low IgG concentrations. Sera from immunocompromised patients were tested with dye-test (DT), LDBio, Platelia and Toxoscreen. The results of DT and LDBio were identical but different from those of Platelia in 90% of cases. These results suggested that a confirmatory assay such as LDBio II is efficient enough to identify specific IgG in the grey zone. Despite its high cost, its implementation is easy compared to DT, which requires time and live *T. gondii* tachyzoites.

In our study, the sensitivity of serological tests was greatly increased when the doubtful results of each assay were considered positive, while maintaining excellent specificity. Murat et al*.* [12] compared Vidas’s performance with that of Liaison and Architect. As in our study, the sensitivity of Liaison (93.8%) remained the same, whether the values of the grey zone were considered positive or negative. For Vidas, the sensitivity increased from 93.8% when the doubtful results were considered negative to 98.4% when they were considered positive. For Architect, the difference was greater; the sensitivity went from 84.4% when they were considered negative to 93.8% when the doubtful results were regarded as positive. In the study by Leslé et al*.* [7], as in our study, the sensitivity of Elecsys was strongly increased to (98.5%) when the results in the grey zone were considered positive and that of Platelia (16.3%) remained unsatisfactory. The threshold of positivity of these techniques could thus be lowered for immunocompromised patients, allowing for better detection of seropositive patients with a low level of IgG. The entry threshold in the grey zone could also be decreased, making it possible to redefine a grey zone more adapted to immunocompromised patients. For most assays, the LDBio II performed on the sera with results in the supplier’s grey zone was always positive, except for Elecsys.

In our study, new thresholds were proposed for Architect, Platelia and Elecsys. Two studies were conducted on the reduction of positivity thresholds for *T. gondii* IgG. Leslé et al*.* [7] proposed a modification of the positivity threshold for Platelia. With a positivity threshold of 6 IU/mL, 90.5% of the results were negative, while the WB was positive for 54% of these sera. As in our study, all WBs performed on sera in the grey zone were positive. If the Platelia positivity threshold was lowered to 4 IU/mL, then the specificity of the test remained at 100%, but the sensitivity was 44%. The sensitivity was significantly lower than that reported by the manufacturers, but the comparison was difficult because the sera did not come from comparable groups. In the study by Mouri et al*.* [11], 384 positive or equivocal IgG sera with Platelia were selected, of which 48 were from immunocompromised patients. All serologies were confirmed with LDbio. Of the 261 equivocal sera with Platelia, 244 were positive with WB. By modifying the positivity threshold of Platelia to 4.4 IU/mL, 30.7% of the equivocal or negative sera would be made positive in agreement with the WB. In the immunocompromised group, this threshold change made it possible to have a sensitivity of 42% and a specificity of 100%. For immunocompromised patients, this threshold would reduce the number of confirmatory tests. The positivity thresholds proposed in these two publications were similar to those chosen in our study for Platelia. Unfortunately, no grey zone threshold was proposed, which would trigger a confirmatory test.

In studying the positive results of LDBio II, the profile showing bands at 30–31–33 associated with other bands or not was most often found. This profile could be a sign of old immunity. In the negative WBs, some had only band 30. In the study by Maudry et al. [9], the WB was considered doubtful if band 30 was present but there were less than three bands. In the study by Franck et al*.* [4], the only serology made positive with DT and negative with LDBio II had a single band at 30. Moreover, in studies assessing the early detection of IgG during seroconversion, band 30 is often the first to be highlighted [3, 5]. Band 30 could be a sensitive and specific marker for the presence of low level IgG, that is to say, not allowing us to exclude old or recent contact with *T. gondii*. In our study, the insufficient volume of serum did not make it possible to carry out the dye tests on the sera with doubtful results with WB.

Waiting for an official change of the thresholds, an evaluation in a multicentric prospective study of immunodeficient patients could help refine the calculations of sensitivities and specificities by including higher levels of IgG. As of now, users of these reagents in immunocompromised patients should be alerted to the possible presence of *Toxoplasma gondii* in case of non-zero levels of IgG. Given the risk of clinical reactivation of the parasite in these patients and the availability of prophylactic treatment, it seems essential to implement a more sensitive confirmation technique (WB or DT) or to transmit the serum to an expert centre to conclude.

A study on the re-evaluation of these thresholds could also be conducted in pregnant women, who sometimes have low IgG levels, and the IgG levels may be made negative by the automated systems, resulting in unnecessary monthly monitoring. Finally, it would be interesting to perform a DT on “doubtful” WB to evaluate whether in the presence of a band with less than three bands, in immunocompromised patients, the result of LDBio II can be considered doubtful, that is, not permitting exclusion of contact with *T. gondii.*

## Conflict of interest

This research did not receive any specific grant from funding agencies in the public, commercial, or not-for-profit sectors.
